# On the Non-Stationary Relationship between the Siberian High and Arctic Oscillation

**DOI:** 10.1371/journal.pone.0158122

**Published:** 2016-06-30

**Authors:** Wenyu Huang, Bin Wang, Jonathon S. Wright, Ruyan Chen

**Affiliations:** 1 Ministry of Education Key Laboratory for Earth System Modeling, Center for Earth System Science, Tsinghua University, Beijing, China; 2 State Key Laboratory of Numerical Modeling for Atmospheric Sciences and Geophysical Fluid Dynamics, Institute of Atmospheric Physics, Chinese Academy of Sciences, Beijing, China; University of California San Diego, UNITED STATES

## Abstract

An area-weighted *k*-means clustering method based on pattern correlations is proposed and used to explore the relationship between the Siberian High (SH) and Arctic Oscillation (AO) during the winter months (December-January-February) of 1948–2014. Five regimes are identified. Four of these five regimes (comprising 171 of 201 months) show a negative correlation between the SH and AO indices, while the last regime (30 months) shows a positive correlation. The location of the SH shifts southward into China under two of the four negative-correlation regimes (117 months), with pressure variations over the center of activity for the SH opposite to pressure variations over the climatological center of the SH (which is used to define the SH index). Adjusting the SH index to account for these spatial shifts suggests positive rather than negative correlations between major variations in the SH and AO under these regimes. Under one of the two remaining negative-correlation regimes, pressure anomalies are weak over the Arctic Ocean. In total, only one regime comprising 21 of 201 months strictly obeys the negative correlation between the SH and AO reported by previous studies. The climate regime characterized by an intensified SH is associated with a greater frequency of cold surges over northern and southeastern China, and the weakening of the East Asian winter monsoon during the 1980s was accompanied by a sharp reduction in the occurrence of this regime.

## Introduction

The Siberian High (SH) is a surface high-pressure system that covers large portions of the Eurasian continent during wintertime [1–3]. The SH is one of the major components of the East Asia winter monsoon (EAWM), and its variability is closely linked to the variability of cold surges in East Asia [1, 4, 5]. Given the geographical influence and long duration of the SH, it is important to consider the coupling between the SH and wintertime Arctic Oscillation (AO).

The AO is the leading mode of climate variability in the Northern Hemisphere [6, 7]. Gong et al. [8] reported the existence of a significant negative correlation between the SH and AO during the period 1958–1998; however, Wu and Wang [9] pointed out that the SH and AO time series varied out of phase and were even positively correlated over some periods. The complicated relationship between AO and SH limits the use of AO variability as a potential indicator for changes in the intensity of the SH and EAWM.

In climate science, Empirical Orthogonal Function (EOF) analysis is the most frequently used approach for deriving major spatial patterns of variability and their evolution in time from 3-dimensional spatiotemporal data. One of the biggest weaknesses of this approach is that the positive and negative polarities for each extracted spatial pattern are assumed to be symmetric [10]. The AO index used for studying connections between the AO and SH is based on EOF analysis [11]. Moreover, the most frequently used index for the SH is defined as an area average of sea level pressure over the climatological center of activity for the SH [8], which means that this index cannot effectively distinguish variations in the intensity of the SH from variations in the location of the SH. These shortcomings limit and potentially bias our understanding of the coupled relationship between the SH and AO.

Clustering analysis [12] can provide a complementary classification-based perspective that overcomes the aforementioned shortcoming in the EOF-based approach. It has been used successfully to detect climate regimes over the North Atlantic [13] and wintertime circulation regimes over North America [10]. However, clustering is less popular than EOF analysis among the climate research community, due mainly to the following two reasons. First, most clustering algorithms extract centroids based on distances between samples and centroids [12, 14–16]. Most climate studies focus on anomalies relative to a mean field, and prefer to classify anomalies into categorical types according to their phase (positive or negative) rather than their amplitude (as distance-based clustering methods do). Second, most climate data are archived on longitude–latitude grids. The majority of clustering methods are not designed for such grids, which leads to over-weighting of anomalies at high latitudes.

In this study, we design a new clustering method for use with longitude–latitude anomaly fields, and then apply it to explore the connection between the SH and AO. The paper is organized as follows. We introduce the underlying data and the new clustering method in section 2. We then discuss the non-stationary relationship between the SH and AO revealed by the clustering method in section 3, and use these results to provide new insight on variability in the EAWM and cold surges over Eurasia. We summarize the conclusions of this work in section 4.

## Data and Methods

### Data

We use the National Centers for Environmental Prediction and the National Center for Atmospheric Research (NCEP-NCAR) reanalysis data [17] from December 1948 to February 2015. The variables taken from the NCEP-NCAR data include monthly sea level pressure (SLP), air temperature and winds on the pressure levels, monthly surface winds at 10-m height above surface, and daily minimum temperature at 2-m height above surface. We also use a Niño3 index based on the Hadley Centre Sea Ice and Sea Surface Temperature analysis version 1 (HadISST1) [18] to represent variability in the El Niño–Southern Oscillation (ENSO). In this study, the winter for each year refers to December of that year and January–February of the following year (DJF).

### Description of the clustering method

The new clustering method is designed for application to anomaly fields on longitude–latitude grids. The main basis of the method is the calculation of area-weighted spatial pattern correlation coefficients (Area-Weighted-PC) between paired spatial patterns. The Area-Weighted-PC is estimated using the formula

$$\text{Area-Weighted-PC}=\frac{\sum_{i=1}^{n}\sum_{j=1}^{m}W_{i,j}X_{i,j}Y_{i,j}}{{\left(\sum_{i=1}^{n}\sum_{j=1}^{m}W_{i,j}X_{i,j}^{2}\right)}^{\frac{1}{2}}{\left(\sum_{i=1}^{n}\sum_{j=1}^{m}W_{i,j}Y_{i,j}^{2}\right)}^{\frac{1}{2}}}\;,$$ (1)

where (*X*, *Y*) is a pair of spatial patterns, (*i*, *j*) are the indices for longitude and latitude, respectively, (*n*, *m*) are the numbers of grid points in the longitude and latitude directions, respectively, and *W* is an area weight coefficient that varies by location. The value of Area-Weighted-PC ranges from -1 to 1. The absolute magnitude of Area-Weighted-PC increases with the similarity of the patterns *X* and *Y*. Negative values indicate anticorrelation rather than dissimilarity.

Given *N* samples from which we wish to derive *M* clusters, the clustering method can be summarized as follows. First, we calculate Area-Weighted-PCs between all possible pairs of samples, and use these Area-Weighted-PCs to define *M* initial centroids. For every sample, we evaluate the number of Area-Weighted-PCs with the other *N*–1 that exceed a critical threshold for Area-Weighted-PC (a value ranges from 0.3 to 0.8). The first initial centroid is identified as the sample with the largest number of Area-Weighted-PCs exceeding the critical threshold. This number is then defined as *LN1*. The second centroid is found using the same method as the first centroid, but after removing the *LN1+1* samples associated with the first centroid. These steps are iterated until *M* initial centroids are found. The *N* samples are then classified into *M* clusters by assigning each sample to the centroid with which it has the largest Area-Weighted-PC. We define the metric Sample Area-Weighted-PC as the Area-Weighted-PC between a sample and its corresponding centroid. The average Sample Area-Weighted-PC over the *N* samples is then used as an indicator for the effectiveness of the clustering method. The *M* centroids are then updated by averaging the samples in each cluster, and *M* new clusters are calculated. The difference in average Sample Area-Weighted-PC between the new clusters and the previous clusters is calculated, and the centroid update step is repeated iteratively until this difference is small. Here, we use 10^−6^ as the criterion for deciding whether further updates are required.

Two key parameters govern the performance of the method, namely the number of the clusters *M* and the critical threshold for the Area-Weighted-PC. In this study we apply a grid search technique to identify optimal value for each parameter. Values of *M* ranging from 2 to 6 and values of the critical threshold for Area-Weighted-PC ranging from 0.3 to 0.8 (with an interval of 0.05) are used to test the sensitivity of the results to various choices of parameters and identify their optimal values. The metric average Sample Area-Weighted-PC is used to evaluate the overall quality of the classification.

## Results and Discussion

### Relationship between the SH and AO as revealed by cluster analysis

**Fig 1:**
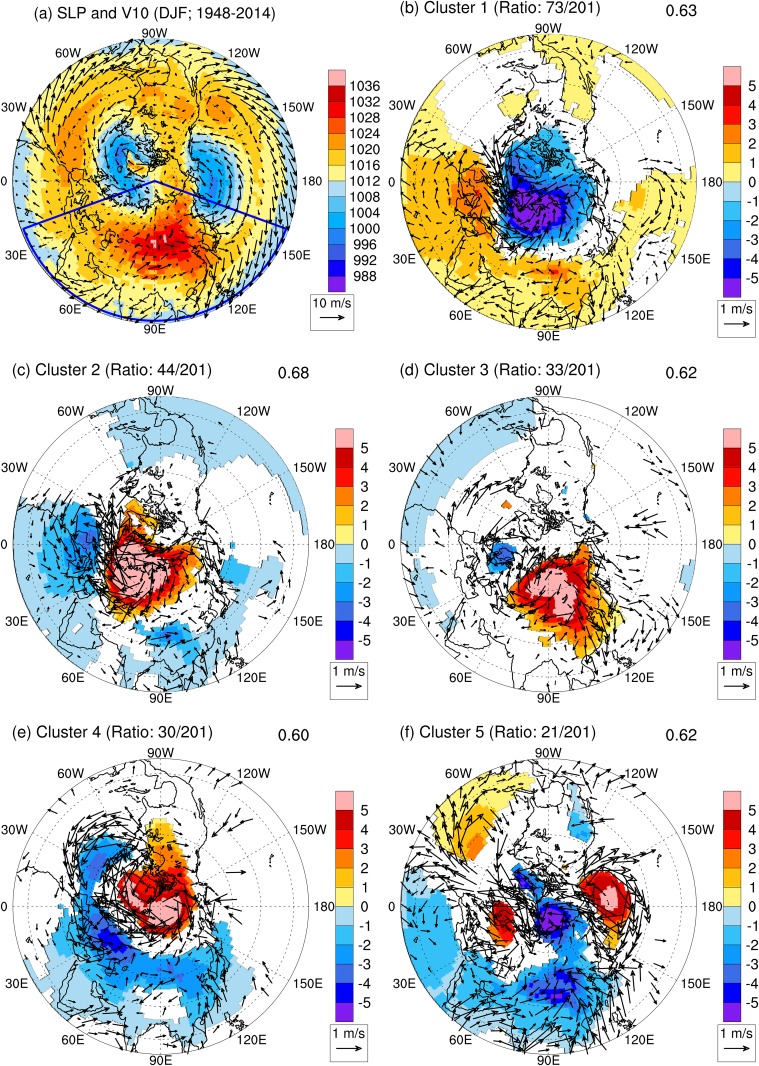
DJF climatology of SLP and surface winds and composite mean monthly anomalies of SLP and surface winds over the Northern Hemisphere associated with each cluster. a. DJF-mean SLP (shading; hPa) and surface winds (vector; m s^–1^) at 10-m height above surface. The area within the blue lines indicates the region (20–160°E, 10–90°N) under consideration by the clustering method. b-f. Composite mean monthly anomalies of SLP and surface winds for each cluster. Statistical significance is evaluated using the 95% confidence level based on the Student’s *t* test. Composited SLP anomalies are plotted if they are significant. Wind anomalies are plotted if wind anomalies are significant in at least one direction (east–west or north–south). The ratio of the number of samples belonging to each regime to the total number of samples is included at the top left of each panel. The average spatial pattern correlation coefficient for each regime is shown separately at the top right corner of each panel. All quantities are based on the NCEP-NCAR reanalysis from December 1948 through February 2015.

Fig 1a shows the long-term climatological mean winter SLP and surface winds over the Eurasian continent during 1948–2014. The distribution of SLP over this region is dominated by a pronounced surface high-pressure system centered over north–central Asia: the SH. The climatological center of the SH has a maximum SLP of 1039.39 hPa, located to the west of Lake Baikal. Strong near-surface northerly winds along the east coast of the Eurasian continent bring cold air from high latitudes to low latitudes, so that temperatures there are colder relative to the zonal mean (not shown). The climatological wintertime SH is bracketed by the Aleutian low to its east and the Icelandic low to its west (Fig 1a).

The Area-Weighted-PC-based *k*-means clustering algorithm is employed to identify different climate regimes (clusters) of SLP over Eurasia via the following two steps. First, the winter climatology of SLP (the long-term DJF mean) during 1948–2014 (Fig 1a) is removed from monthly mean SLP during DJF, yielding 201 (*N* = 67 years × 3 months) monthly anomalies for SLP. Note that the subtracted climatology of SLP is the same for all the winter months, including December, January, and February. Second, the clustering algorithm is applied to monthly pressure anomalies over the Eurasian continent and surrounding oceans (20–160°E, 10–90°N; hereafter referred to as the regime area) to identify a set of *M* clusters. We examine the sensitivity of the clustering results to various choices of the parameter *M* and the critical threshold for Area-Weighted-PC. Table 1 lists the average Sample Area-Weighted-PC for all samples as a function of *M* and the critical threshold for Area-Weighted-PC, while Table 2 lists the minimum of the averages of the Sample Area-Weighted-PC for the *M* clusters as a function of *M* and the critical threshold for Area-Weighted-PC. Both the average Sample Area-Weighted-PC for all samples and the minimum of the averages of the Sample Area-Weighted-PC for the *M* clusters typically increase with *M*, with only a few exceptions (see Table 2). However, larger values of M can overcomplicate further analysis of the regimes by highlighting progressively smaller differences that play minor roles in the large-scale circulation, and are therefore undesirable. To ensure the quality of the clustering classification while limiting the number of clusters, we set a critical threshold of 0.6 for the minimum of the averages of the Sample Area-Weighted-PC for the *M* clusters. Based on this criterion, the optimal parameter values are *M* = 5 and the critical threshold for Area-Weighted-PC = 0.55, yielding a minimum of the averages of Sample Area-Weighted-PC of 0.60 for the *M* clusters (Table 2). The average pattern correlation for every cluster exceeds at least 0.6 under this combination of parameters. The corresponding average Sample Area-Weighted-PC for all samples is 0.64 (Table 1).

**Table 1:** The average Sample Area-Weighted-PC for all samples for applications of the clustering algorithm to DJF SLP anomalies using different values of the critical threshold for Area-Weighted-PC (0.3–0.8) and *M* (2–6). To keep consistence with the criterion (10^−6^) for iteration during clustering, results are presented with 6 digits.

Critical threshold	*M* = 2	*M* = 3	*M* = 4	*M* = 5	*M* = 6
**0.3**	0.502830	0.548165	0.599375	0.631463	0.651947
**0.35**	0.502830	0.567451	0.605702	0.635706	0.649095
**0.4**	0.502830	0.567578	0.604703	0.627313	0.653231
**0.45**	0.502830	0.567291	0.604806	0.623018	0.640724
**0.5**	0.502830	0.566611	0.603860	0.635600	0.644347
**0.55**	0.502847	0.566396	0.603319	0.637058	0.658667
**0.6**	0.502830	0.566759	0.597342	0.627883	0.654272
**0.65**	0.502847	0.556093	0.597377	0.627357	0.649956
**0.7**	0.502847	0.556093	0.603284	0.627185	0.651531
**0.75**	0.502830	0.556093	0.580383	0.618990	0.653414
**0.8**	0.502830	0.556093	0.595293	0.634233	0.652030

**Table 2:** The minimum of the averages of Sample Area-Weighted-PC for the *M* clusters under different values of the critical threshold for Area-Weighted-PC (0.3–0.8) and *M* (2–6). Due to the same reason given in Table 1, data are presented with 6 digits.

Critical threshold	*M* = 2	*M* = 3	*M* = 4	*M* = 5	*M* = 6
**0.3**	0.488246	0.531226	0.554818	0.596295	0.606109
**0.35**	0.488246	0.546634	0.546771	0.587312	0.601664
**0.4**	0.488246	0.519394	0.540843	0.534142	0.597696
**0.45**	0.488246	0.528112	0.573705	0.590214	0.613969
**0.5**	0.488246	0.507268	0.581114	0.571365	0.596356
**0.55**	0.483529	0.526676	0.545872	0.603670	0.614108
**0.6**	0.488246	0.507268	0.529393	0.582666	0.601516
**0.65**	0.483529	0.525810	0.590423	0.586959	0.586959
**0.7**	0.483529	0.525810	0.537852	0.574686	0.620884
**0.75**	0.488246	0.525810	0.549434	0.590407	0.595715
**0.8**	0.488246	0.525810	0.582385	0.597281	0.597208

**Fig 2:**
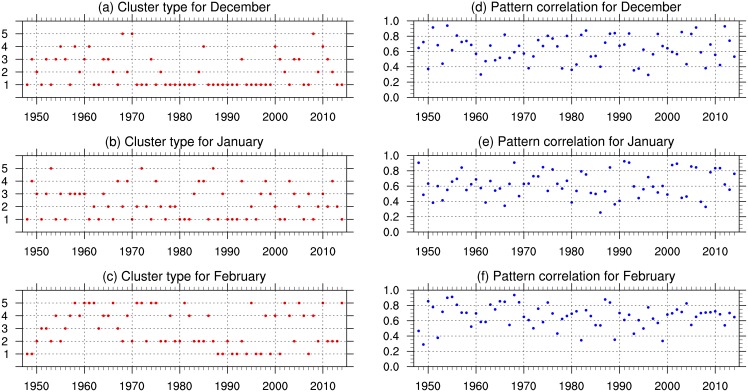
Regime type and area-weighted pattern correlation coefficient for each month during DJF 1948–2014. The pattern correlation coefficients shown in panels d–f are calculated between the monthly anomalies and their corresponding regimes.

The composited means of SLP anomalies and 10-m near-surface wind anomalies for the five regimes are shown in Fig 1b–1f. Fig 2 tracks the regime type identified for each month during DJF of 1948–2014 (Fig 2a–2c), along with the pattern correlation between each month and its corresponding regime (Fig 2d–2f). The number of months corresponding to each regime can be used to rank the occurrence frequencies of the five regimes during 1948–2014. Regime 1 (cluster 1; Fig 1b), which comprises 73 months (about 36.32% of the total number of months), is dominated by negative SLP anomalies in high latitudes (>45°N) peaking over the northwest coast of the Eurasian continent, and positive anomalies in low and mid latitudes (< 45°N) peaking in central China. Regime 2 (Fig 1c), which comprises 44 months (21.89%), features positive pressure anomalies in high latitudes (north of 45°N) peaking over the northwest coast of the Eurasian continent, and negative anomalies in low and mid latitudes (south of 45°N) peaking over central China; this cluster contrasts with regime 1. In both of these regimes, the center of activity for the SH is located over central China. For example, regime 1 reflects an intensified southern portion of the SH, which leads to stronger northerlies along the southeastern (south of 25°N) coast of China. By contrast, regime 2 reflects a weakened southern portion of the SH, which leads to weaker northerlies along the southeastern coast of China.

Regime 3 (Fig 1d), which comprises 33 months (16.42%), features a systematic intensification of the SH. High SLP within the SH is associated with strong northerlies along the east coast of the Eurasian continent, which are the strongest northerly anomalies among the five regimes. Regime 4 (Fig 1e), which comprises 30 months (14.93%), features a reduction of pressure over most of the Eurasian continent, with a few exceptions along the coast of the Arctic Ocean. This reduction in SLP over the continent is accompanied by a significant increase in SLP over the Arctic Ocean. Regime 5 (Fig 1f), which comprises 21 months (10.45%), is characterized by an evident reduction in SLP over most parts of the Eurasian continent, with the exception of the northwestern quadrant. Unlike regime 4, regime 5 includes a significant reduction in SLP over the Arctic Ocean.

Two indices are adopted to enable quantitative study of the relationship between the AO and SH. Following Wu and Wang [9], the SH intensity index is defined as the average of SLP over the climatological center of the SH (80–120°E, 40–60°N). The AO index is defined as the principal component time series of the leading EOF of monthly SLP anomalies over regions north of 20°N [11]. The AO is often treated as equivalent to the North Atlantic Oscillation (NAO); however, Ambaum et al. [19] showed that covariability in surface pressure among all of the centers of action are captured more effectively by the NAO index than by the AO index. We therefore also use the NAO index here to supplement the AO index in describing pressure anomalies in high latitudes. The NAO index is calculated similarly to the AO index, but within the Atlantic sector (90°W–40°E, 20–80°N) [20, 21]. The time series for all three indices during the winter months are normalized to have a mean of zero and a standard deviation of one during 1948–2014. As expected, the time series of monthly AO index during the winter months of 1948–2014 is highly correlated with that of the NAO index (*R* = 0.92).

The linear correlation coefficient between the AO and SH is –0.25 (significant at the 99% confidence level; 199 degrees of freedom), consistent with previous results [8, 22]. We calculate composite mean monthly SH, AO, and NAO indices for each climate regime (Table 3). The significance of each composite mean index is tested against the composite mean for the complementary set of months using a two-sided Student’s *t* test. The SH index varies considerably across regimes, with differences that are consistently significant at the 99% confidence level except for that associated with regime 2 (95% confidence level). Significant changes can also be identified in the AO index, with all changes significant at the 99% confidence level except for that associated with regime 3 (90% confidence level), when the mean SH is more than one standard deviation above the climatological mean. Regime 3 features the smallest absolute value of the AO index and an NAO index close to zero, reflecting the weak pressure anomalies over the North Pole and Arctic Ocean, particularly in the Atlantic sector (Fig 1d). Four of the five regimes indicate an anti-correlation between the SH and AO, with the exception of regime 4 (Table 3). Regime 4 includes a prominent high pressure anomaly over the Arctic Ocean (i.e., the negative phase of the AO), but this high pressure anomaly is restricted to the Arctic Ocean and affects only the northernmost flank of the SH (Fig 1e). Pressure drops over major parts of the SH domain during regime 4 (i.e., the negative phase of the SH), contrary to the expected negative correlation between the SH and AO.

**Table 3:** Composite means of climate indices for different SLP anomaly regimes.

	SH	AO	NAO	NP	Niño3
Cluster1	-0.28***	0.71***	0.51***	0.03	0.15**
Cluster2	0.29**	-0.77***	-0.57***	-0.22*	-0.08
Cluster3	1.35***	-0.28*	0.00	-0.14	-0.30*
Cluster4	-0.65***	-0.69***	-0.61***	-0.02	-0.24
Cluster5	-0.80***	0.58***	0.30	0.59***	-0.04

One, two and three asterisks (*, ** and ***) indicate that the indices exceed the 0.1, 0.05 and 0.01 significance levels, respectively. Statistical testing is based on two-sided Student’s t test against the complementary set of months (i.e., the months not belonging to the specified cluster).

The SH and AO are anticorrelated in regimes 1 and 2; however, the composite mean SH indices during these two regimes are smaller in absolute magnitude than those associated with the other three regimes (Table 3). This weakness reflects shifts in the location of the SLP anomaly relative to the climatological center of the SH: the SLP anomaly is centered over the northwest coast of Eurasia, with the climatological center of the SH located to its southeast (Fig 1b and 1c). Under these two regimes, the SLP anomaly at the climatological center of the SH has the same sign as the SLP anomaly over the Arctic Ocean, with a negative pattern correlation between regime 1 and regime 2. Takaya and Nakamura [23] pointed out that an enhanced SH amplifies the wintertime land–sea pressure contrast and typically induces northerly anomalies along the east coast of China. However, the monsoonal northerlies do not increase with the intensity of the SH index in these two regimes; in contrast, the northerlies over the southeastern coast decrease with the SLP over the climatological center of the SH. The center of action for the SH is located over central China under both regime 1 and regime 2, far from climatological center of the SH. A new index reflecting changes in both the intensity and the center of action for the SH would establish a positive correlation between the SH and AO, in contrast to the negative correlation found when using the traditional SH index.

The negative correlation between the AO and SH is well established in regime 5, which corresponds to an anomalously weak SH associated with the positive phase of the AO. The monsoonal northerlies are significantly reduced due to the weaker SH (Fig 1f). The relationship between the AO and SH revealed by the clustering analysis is therefore rather complicated. Although four of the five regimes (including 171 months) reveal the expected negative correlations when traditional indices are used to represent the SH and AO, the regime most typical of the expected relationship (regime 5) includes only 21 months.

We further examine the relationships between the SH and the Aleutian Low and between the SH and the ENSO during each regime. The intensity of the Aleutian Low is represented by the North Pacific index (NP), defined as the area-mean SLP over 160°E–140°W and 30–65°N [24]. ENSO variability is represented by the Niño3 index based on HadISST1 data [18]. As with the other indices, the NP index for the winter months of 1948–2014 is normalized to have a mean of zero and a standard deviation of one. Since the data for Niño3 index available to us has been normalized, a further normalization is not taken.

The loading pattern of the AO (the leading EOF of SLP northward of 20°N) indicates that SLP anomalies associated with the Aleutian Low vary in the opposite phase to those in the Arctic Ocean, which implies a positive correlation between the NP and AO indices [25]. Visual inspection reveals that this positive correlation is well captured by regimes 1, 2 and 5 (Fig 1b, 1c and 1f), for which the linear correlation coefficient is 0.26 (significant at the 99% confidence level). However, only the composite mean NP index for regime 5 exceeds the 99% confidence level. During regime 5, southerly anomalies along the eastern coast of the Eurasian continent induced by the reduced Aleutian Low reinforce the weakening of the SH and monsoonal northerlies (Fig 1f). Most regimes do not include strong preferences for the positive or negative phases of ENSO. Regime 1 is associated with a composite mean Niño3 index of 0.15 that is significant at the 95% confidence level; however, this index is too small to conclusively link this regime to the positive phase of ENSO. Regime 3 is associated with a mean Niño3 index of –0.30 that is significant at the 90% confidence level, as well as the largest SH index (1.35; 99% confidence). Previous studies have suggested that the intensified SH during the negative phase of ENSO can be partially explained by the Pacific–East Asia teleconnection [26], which generates a cyclonic anomaly in the lower troposphere over the North Pacific during La Niña years. This cyclonic anomaly favors the southward displacement of the SH and an accompanying southward incursion of cold air. The southward incursion of cold air induces an anti-cyclonic anomaly, which reinforces and intensifies the positive anomaly in the SH.

### A comparison with two existing clustering methods

Two commonly used clustering methods, i.e., the distance-based *k*-means clustering and Self-Organizing Map (SOM), are adopted here to make a comparison with the Area-Weighted-PC-based *k*-means clustering method. Both of the two clustering methods are applied to the same data under consideration by the Area-Weighted-PC-based *k*-means clustering method, i.e., the winter monthly SLP anomalies during 1948–2014 over the region of 20–160°E, 10–90°N. The clusters in SOM are connected to the adjacent clusters via the rectangular topology. Table 4 lists the average Sample Area-Weighted-PC for all samples as a function of the number of clusters *M*, which ranges from 2 to 6. It is worth noting that there are two possible SOM grids when the number of clusters *M* equals 4 or 6. When *M* is set to 4, the SOM grids could be 1×4 and 2×2. When *M* is set to 6, the SOM grids could be 1×6 and 2×3. The results of the Area-Weighted-PC-based *k*-means method for a specified *M* are estimated as the column-averages of Tables 1 and 2, i.e., the averages of the results for different values (from 0.3 to 0.8 with an interval of 0.05) of the critical threshold for the Area-Weighted-PC. The average Sample Area-Weighted-PC of all samples for both the distance-based *k*-means and SOM methods increase with the number of clusters *M*, which are similar to those of the Area-Weighted-PC-based *k*-means method. For all the values of *M* (2–6), an increase ranging from 0.02 to 0.07 can be identified in the average Sample Area-Weighted-PC of all samples based on the Area-Weighted-PC-based *k*-means method, compared to those based on the distance-based *k*-means and SOM methods. However, in both the distance-based *k*-means and SOM methods, the minimum of the averages of Sample Area-Weighted-PC for the *M* clusters does not increase with the number of the clusters *M* (Table 5). Evident improvements can also be identified in the minimum of the averages of Sample Area-Weighted-PC based on the Area-Weighted-PC-based *k*-means method, compared to those based on the distance-based *k*-means and SOM methods, for all the values of *M* (ranging from 2 to 6) except for *M* = 3 at which the minimum of the averages of Sample Area-Weighted-PC for both the Area-Weighted-PC-based *k*-means and SOM methods equals 0.52. When the number of clusters *M* equals 5, the minima of the averages of Sample Area-Weighted-PC for the *M* clusters are 0.49 and 0.47 for the distance-based *k*-means and SOM methods, respectively, which are significantly less than the value (0.58) for the Area-Weighted-PC-based *k*-means method.

**Table 4:** The average Sample Area-Weighted-PC for all samples for the *M* clusters under different values of *M* (2–6) based on the distance-based *k*-means, Area-Weighted-PC-based *k*-means, and SOM clustering methods. Note that result for the Area-Weighted-PC-based *k*-means method is estimated as the column-average of Table 1. In the SOM, when the number of clusters is set to 4 or 6, there are two possible configurations of the SOM grids, i.e., 1×4 and 2×2 for *M* = 4, and 1×6 and 2×3 for *M* = 6. Data are presented with 2 digits.

	Distance-based *k*-means	Area-Weighted-PC-based *k*-means	SOM			
***M* = 2**	0.48	0.50	1x2	0.48	/	/
***M* = 3**	0.51	0.56	1x3	0.54	/	/
***M* = 4**	0.56	0.60	1x4	0.55	2x2	0.55
***M* = 5**	0.59	0.63	1x5	0.57	/	/
***M* = 6**	0.62	0.65	1x6	0.58	2x3	0.60

**Table 5:** Same as Table 4, except for the minimum of the averages of Sample Area-Weighted-PC for the *M* clusters. Note that result for the Area-Weighted-PC-based *k*-means method is estimated as the column-average of Table 2.

	Distance-based *k*-means	Area-Weighted-PC-based *k*-means	SOM			
***M* = 2**	0.46	0.49	1x2	0.45	/	/
***M* = 3**	0.35	0.52	1x3	0.52	/	/
***M* = 4**	0.52	0.56	1x4	0.47	2x2	0.48
***M* = 5**	0.49	0.58	1x5	0.47	/	/
***M* = 6**	0.54	0.60	1x6	0.31	2x3	0.52

### Cold surges under different regimes

Cold surges are among the main climate-related disasters during winter months over the Eurasian continent [4, 5, 27]. Here, we discuss how the distribution and duration of cold surges over Eurasia change under the five SH regimes identified by the cluster analysis. We adopt the cold spell duration index (CSDI) [28] to represent the temporal and spatial variations in cold surges. The CSDI is defined as the monthly count of days included in stretches of at least five consecutive days with daily minimum surface air temperatures at 2-m height below the 10^th^ percentile for that 5-day calendar window. The 10^th^ percentile is defined relative to the base period 1961–1990. Days belonging to a cold surge event that spans two months are apportioned to their respective calendar months.

**Fig 3:**
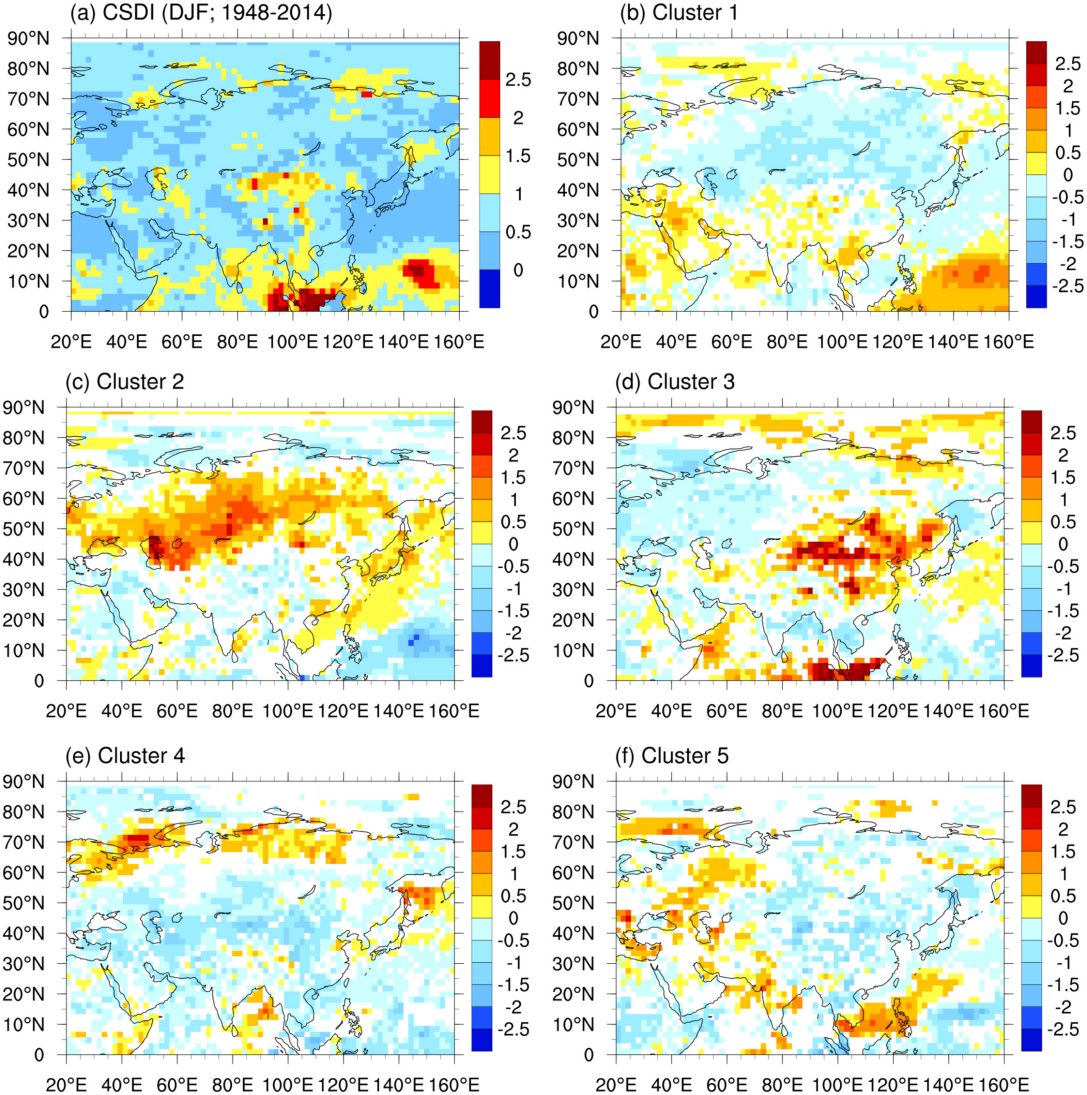
DJF Climatology of CSDI (a; units: days) and composited means (b-f) of monthly CSDI anomalies for different regimes during DJF over 1948–2014. In b-f, only composited means that meet the 95% confidence level of the *F* test are plotted.

Fig 3a shows the spatial distribution of the long-term climatological mean CSDI for winter months during 1948–2014. Climatologically, cold surges occur most frequently in northern and central China. Monthly anomalies of the CSDI are obtained by removing this climatology. Fig 3b–3f shows composited means of monthly anomalies of the CSDI for the five SH regimes identified using the clustering method (Fig 1b–1f). Mean values of the CSDI associated with regimes 4 and 5 are similar to or smaller than the climatological mean CSDI over most parts of Eurasia. We therefore use a variance ratio-based test method (the two-sided *F* test) to test the significance of the CSDI anomalies associated with each regime.

Under regime 1 (Fig 3b), the CSDI is reduced to near zero over large portions of Siberia. This change can be explained by a reduction in the occurrence of cold air outbreaks from polar regions due to southerly anomalies in near-surface winds (Fig 1b). Despite the high SLP anomaly along the southern edge of the SH, the CSDI over China does not increase substantially. This lack of increase can be attributed to the reduction in cold surges in Siberia. Under regime 2 (Fig 3c), the CSDI over Siberia increases remarkably due to enhanced northerlies over this region (Fig 1c). Anomalously low SLP along the southern flank of the SH constrains this increase to high latitudes, leaving the CSDI slightly reduced over most parts of China. Under regime 3 (Fig 3d), the powerful northerlies associated with the sharply enhanced SH result in dramatic increases in the CSDI over northern and southeastern China. Under regimes 4 and 5 (Fig 3e and 3f), the CSDI is reduced over most parts of China. This change is mainly attributable to the weaker monsoonal northerlies associated with the reduced intensity of the SH. By contrast, the enhanced northerlies over the north coast of Siberia under regime 4 and the enhanced northerlies over western Eurasia under regime 5 result in significant increases in the CSDI over these regions (Fig 3e and 3f).

### Relationships with variability in the EAWM

**Fig 4:**
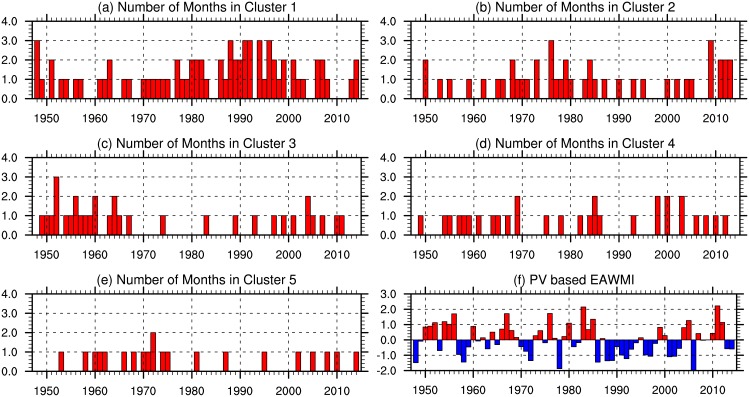
Time series of the number of DJF months belonging to each regime (a-e) and the normalized DJF-mean PV-based EAWM index (f). The EAWM index is normalized to have a mean of zero and a standard deviation of one during 1948–2014.

In this section, possible connections between different SH regimes and variability in the EAWM are briefly explored. An isentropic potential vorticity (PV) intrusion-based EAWM index [29] is adopted, calculated as

$$I_{EAWM}=\bar{PV_{300K}(90-150^{o}E,20-50^{o}N)}-\bar{PV_{300K}(0-360^{o}E,20-50^{o}N)}\;,$$ (2)

where the overbar indicates an area average, the first term on the right side is the area-mean PV at 300 K potential temperature level over the East Asia (90–150°E, 20–50°N), and the second term is the area-mean PV at 300 K averaged over the entire 20–50°N latitudinal band. This index can be considered as the PV anomaly in the East Asia with respect to the zonal mean. This PV-based EAWM index captures the key climatological aspects of the EAWM, including the dynamical relationships between the EAWM and the AO [8], ENSO [26, 30] and SH [22], and the weakening trend in EAWM intensity during the 1980s [31, 32]. The major advantage of this PV-based EAWM index is its physical basis: based on the technique of PV intrusion, a year with stronger PV intrusion will lead to an enhanced SH, intensified northerlies over the coastal regions of East Asia and its surrounding oceans, and more severe cold surge. We calculate the winter (DJF) mean EAWM index (Fig 4f), in contrast to the composited means used above, and evaluate its variability relative to the number of months per year belonging to each of the five SH regimes discussed above (Fig 4a–4e).

Linear correlations between the DJF-mean EAWM index and the count of months in each regime are –0.49 for regime 1 (99% confidence), 0.34 for regime 2 (99% confidence), 0.37 for regime 3 (99% confidence), –0.02 for regime 4, and –0.10 for regime 5. Variations in the EAWM are closely related to regimes 1, 2 and 3, which suggests that occurrences of regimes 1, 2 and 3 can be good indicators for variability in the EAWM. Among the most evident features in the time series of the EAWM index is the continued weakening after 1985. Before 1985, the average occurrence frequencies of regimes 2 and 3 were 0.70 and 0.59 months per year, while the average occurrence frequency of regime 1 was 0.89 months per year. During 1985–2000, the average occurrence frequencies of regimes 2 and 3 were decreased markedly to only 0.38 and 0.25 months per year, while that of regime 1 was increased significantly to 1.75 months per year. Understanding this shift from regimes 2 and 3 to regime 1 may help to illuminate the mechanisms behind the weakening of the EAWM during the 1980s.

## Conclusions

A new clustering algorithm is designed for use with climate anomaly data on longitude–latitude grids. Applying the clustering method to monthly SLP anomalies during winter, we obtain five climate regimes that reflect variability in the SH. The average pattern correlations between the samples and the centroids for the five regimes all exceed 0.6, indicating that the classification is successful.

The results of the clustering reveal that only a small ratio of the winter months (21 of 201) strictly obey the expected anti-correlation between the SH and AO. The non-stationary relationship between SH and AO based on the five regimes may be more helpful for monitoring and understanding variations in the SH. Different regimes correspond to different likelihoods and locations of cold surges over the Eurasian continent. This framework may therefore help to inform meteorological predictions of cold surges via simple judgments regarding which regime a weather system belongs to. Moreover, the clustering analysis provides a potentially useful perspective on the continuous weakening of the EAWM around the 1980s.
